# Application of two statistical approaches (Bayesian Kernel Machine Regression and Principal Component Regression) to assess breast cancer risk in association to exposure to mixtures of brominated flame retardants and per- and polyfluorinated alkylated substances in the E3N cohort

**DOI:** 10.1186/s12940-022-00840-4

**Published:** 2022-02-26

**Authors:** Pauline Frenoy, Vittorio Perduca, German Cano-Sancho, Jean-Philippe Antignac, Gianluca Severi, Francesca Romana Mancini

**Affiliations:** 1 Paris-Saclay University, UVSQ, Inserm, Gustave Roussy, “Exposome and Heredity” Team, CESP UMR1018, 94805 Villejuif, France; 2 Laboratoire MAP5 (UMR CNRS 8145), Université de Paris, Paris, France; 3 LABERCA, Oniris, INRA, Nantes, France; 4 Department of Statistics, Computer Science, Applications “G. Parenti”, University of Florence, Florence, Italy

**Keywords:** Brominated flame retardants (BFR), Per- and polyfluorinated alkylated substances (PFAS), Breast cancer, Principal Component Regression (PCR), Bayesian Kernel Machine Regression (BKMR)

## Abstract

**Background:**

Brominated flame retardants (BFR) and per- and polyfluorinated alkylated substances (PFAS) are two groups of substances suspected to act as endocrine disruptors. Such substances could therefore be implicated in the occurrence of breast cancer, nevertheless, previous studies have led to inconstant results. Due to the large correlation between these substances, and the possibly non-linear effects they exert, evaluating their joint impact as mixtures on health remains challenging.

This exploratory study aimed to generate hypotheses on the relationship between circulating levels of 7 BFR (6 polybrominated diphenyl ethers and 1 polybrominated biphenyls) and 11 PFAS and the risk of breast cancer in a case–control study nested in the E3N French prospective cohort by performing two methods: Principal Component Regression (PCR) models, and Bayesian Kernel Machine Regression (BKMR) models.

**Methods:**

194 post-menopausal breast cancer cases and 194 controls were included in the present study. Circulating levels of BFR and PFAS were measured by gas chromatography coupled to high-resolution mass spectrometry and liquid chromatography coupled to tandem mass spectrometry, respectively. The first statistical approach was based on Principal Component Analysis (PCA) followed by logistic regression models that included the identified principal components as main exposure variables. The second approach used BKMR models with hierarchical variable selection, this latter being suitable for highly correlated exposures. Both approaches were also run separately for Estrogen Receptor positive (ER +) and Estrogen Receptor negative (ER-) breast cancer cases.

**Results:**

PCA identified four principal components accounting for 67% of the total variance. Component 3 showed a marginal association with ER + breast cancer risk. No clear association between BFR and PFAS mixtures and breast cancer was identified using BKMR models, and the credible intervals obtained were very wide. Finally, the BKMR models suggested a negative cumulative effect of BFR and PFAS on ER- breast cancer risk, and a positive cumulative effect on ER + breast cancer risk.

**Conclusion:**

Although globally no clear association was identified, both approaches suggested a differential effect of BFR and PFAS mixtures on ER + and ER- breast cancer risk. However, the results for ER- breast cancer should be interpreted carefully due to the small number of ER- cases included in the study. Further studies evaluating mixtures of substances on larger study populations are needed.

**Supplementary Information:**

The online version contains supplementary material available at 10.1186/s12940-022-00840-4.

## Background

Polybrominated diphenyl ethers (PBDE) and polybrominated biphenyls (PBB) are two families of brominated flame retardants (BFR) that have been greatly used in a wide range of consumers’ products to reduce their flammability. Although their use has been progressively limited and banned in Europe during the 90 s, due to their resistance to degradation, PBDE and PBB are widespread in the environment [1]. The long-term toxic effects of PBDE and PBB in humans are not completely elucidated, but they are known to have endocrine disrupting properties and in 2019 PBDE have been included in the high-priority list of agents not previously evaluated by the International Agency for Research on Cancer (IARC) Monographs based on relevant bioassay and mechanistic studies [2, 3].

Per- and polyfluorinated alkylated substances (PFAS) are a wide group of synthetic compounds that are water- and oil-repellent and have been used in a large number of industrial and consumer applications [4]. PFAS are characterized by long half-lives in the biota and humans and biomonitoring studies have suggested that PFOA and PFOS, the two main PFAS representatives, are ubiquitously present in the blood of humans worldwide [5]. PFAS are strongly suspected to act as endocrine disrupting chemicals (EDCs), and PFOA has been classified by the IARC as “possibly carcinogenic to humans” (Group 2B) [3, 6].

The incidence of breast cancer has risen in the past decades among Western populations but, despite a large body of research, the etiology has not yet been fully delineated, as established risk factors cannot solely explain this trend [7–9]. There is growing concern that exposure to chemical environmental contaminants, particularly EDCs, could be one of the factors that led to an increased incidence of breast cancer in the Western world [10]. Actually, the impact of mixtures of environmental chemicals along the carcinogenic process can be triggered or boosted by individual chemicals through different mechanisms featuring the key characteristics of carcinogens [11, 12]. For instance, some experimental studies have highlighted a stimulation of human breast cancer cell proliferation, especially for estrogen-sensitive cells, by some PBDE congeners or mixtures [13, 14]. In addition, a study investigating their mechanisms of action in vitro revealed that some PBDE congeners could act as agonists or antagonists of estrogen receptors [15]. Concerning PFAS, studies on human breast cancer cells have also highlighted possible mechanisms of regulations of estrogen receptors by some congeners [16, 17]. Indeed, the possible effect of these substances on breast cancer risk might be different according to the tumor estrogen receptor status. Previous epidemiological studies have attempted to elucidate the relationship between internal exposure levels of EDCs, such as PFAS and BFR, with breast cancer risk, leading to contradictory results [18–29]. In particular, concerning BFR, a previous study conducted in the E3N French prospective cohort using single-pollutant models has identified a non-linear negative association between circulating levels of PBDE-100 and PBDE-153 and breast cancer risk [23]. Concerning PFOS and PFOA, by applying single-pollutant models on data from the E3N cohort data, a positive linear association between circulating level of PFOS and estrogen receptor positive (ER +) breast cancer risks was highlighted, while a positive non-linear association was found between both PFOS and PFOA and estrogen receptor negative (ER-) breast cancer risk [18]. Notably, epidemiological studies have traditionally focused on estimating the health impact of exposure to individual substances not taking into account that in reality people are exposed to complex chemical mixtures. Therefore, estimating the health effects of several concurrent exposures is of increasing interest in epidemiology and has been identified as a research priority by several national and international scientific and advisory organizations [30, 31].

There are several challenges to modeling the effects of exposure to chemical mixtures [32, 33]. Firstly, mixtures components may interact with each other leading to synergistic or antagonistic effects. Secondly, a non-linear exposure–response relationship between exposure to mixtures and health effects may occur, especially in the context of exposure to EDCs. Finally, mixture components are often strongly correlated, leading to large uncertainty in the effect estimates.

Bayesian Kernel Machine Regression (BKMR) is a novel statistical method that addresses the above-mentioned challenges. Indeed BKMR estimates the exposure–response surface accounting for interactions and non-linear relationships by flexibly modeling exposures and it can handle multicollinearity between substances by applying hierarchical variable selection [34–38].

Another classic method used in epidemiology to deal with multicollinearity of multiple exposure variables is Principal Component Analysis (PCA). PCA projects the original data points living in the space of the original, and possibly correlated, variables onto a lower dimensional subspace whose orthogonal axes, referred to as principal components, are uncorrelated, in a way that minimizes the average projection error. Principal components can then be used to predict the outcome of interest by means of classic regression models. [39, 40]. This combination of PCA and regression analysis is referred to as Principal Component Regression (PCR).

The objective of this exploratory study is to generate hypotheses on the relationship between circulating levels of 7 BFR (6 PDBE and 1 PBB) and 11 PFAS and the risk of breast cancer in a case–control study nested in the E3N French prospective cohort using two methods: PCR models, and BKMR models. More specifically, PCR models can highlight profiles of exposures associated with breast cancer, accounting for less informative variables and managing the high dimensionality with orthogonal scores [41]. By construction, these profiles are linear combinations of exposures. Thus by assessing the association of profiles with breast cancer status using logit regression we work under the assumption of linear exposure–response functions. The BKMR approach complements this analysis by identifying predefined groups of substances (variable selection) and by modeling exposure–response functions more flexibly, in particular without assuming their linearity and allowing for interactive effects [33].

## Methods

### The E3N cohort

The E3N study is a large ongoing French prospective cohort of women, set up in France in 1990. The study was approved by the French National Commission for Data Protection and Privacy (ClinicalTrials.gov identifier: NCT03285230); all participants gave written informed consent. The detailed protocol has been described elsewhere [42]. Briefly, 98 995 women born between 1925 and 1950, were included from the French national health insurance plan for people working for the national education system, the *Mutuelle Générale de l'Education Nationale* (MGEN). Women were enrolled in the cohort through a self-administered questionnaire, and were followed by self-administered questionnaires every two years. Detailed cancer risk factor data were collected through questionnaires at different time points during follow-up, including reproductive history, use of hormonal treatments, anthropometric characteristics, smoking habits, alcohol consumption, diet, and physical activity. The average follow-up rate per questionnaire cycle has been of 83%, and to date, the total loss to follow-up since 1990 has been < 3%.

Between 1994 and 1999, E3N participants were invited to donate blood, resulting in the collection of blood samples from approximately 25 000 participants. Each sample was separated into 28 aliquots (i.e. plasma, serum, buffy-coat, leukocytes, and erythrocytes) that were stored in plastic straws in liquid nitrogen containers (− 196 °C) in a biobank.

Breast cancer cases were identified through self-reporting in the questionnaires, from the MGEN files, or through information from death certificates. Deaths were reported by family members and by searches in the MGEN files and causes of death were obtained from the National Death Index. Pathology reports were obtained for the 93% of incident cases. We also considered cases for which pathology reports have not been obtained, because the proportion of false-positive self-reports was low in our study population (< 5%). Cases were identified up to 2013, which was therefore used as the date of end of follow-up in statistical analyses.

### The nested case–control study on breast cancer

As previously described elsewhere [18], we identified 281 breast cancer cases for which at least three aliquots of serum and six aliquots of plasma were available in the biobank. From these, we excluded all cases who had not completed the dietary questionnaire in 1993 (*n* = 27) or who were diagnosed before the blood sampling and/or before the dietary questionnaire (*n* = 11). Cases of Paget’s disease and benign breast disease were also excluded (*n* = 3). Finally, 240 incident breast cancer cases were available. Due to budget constraints, among those, 194 incident postmenopausal breast cancer cases were randomly selected and included in the study. For each case, one control was sampled from women who were free of breast cancer at the time of diagnosis of the corresponding case (density sampling method). Controls were matched to cases by age (± 2 years), menopausal status at blood collection (premenopausal or postmenopausal), body mass index (BMI) at blood collection (< 25 or ≥ 25 kg/m2) and year of blood collection.

### Measurement of biomarkers of exposure

Laboratory analysis of BFR and PFAS have been detailed elsewhere [18, 23]. Briefly, measurements of PFAS in serum were based on a preliminary alkaline digestion followed by a two-stage Solid Phase Extraction purification using polymeric Oasis HLB and graphitized carbon (ENVI-Carb) cartridges, before liquid chromatography coupled to tandem mass spectrometry (LC–MS/MS) measurement. Analysis of BFR involved a liquid/liquid extraction with pentane followed by determinations with gas chromatography (Agilent 7890A) coupled to high-resolution mass spectrometry (GC-HRMS). The quantification was conducted using the isotopic dilution method with ^13^C labeled analogous as internal standards. All the analyses have been conducted in an ISO 17025:2005 accredited laboratory. Serum and plasma lipids were determined enzymatically (Biolabo, Maizy, France). The following 7 BFR and 18 PFAS were measured: 2,2',4,4',5,5'-hexabromobiphenyl (PBB-153), 2,2',4,4',6-pentabromodiphenyl ether (PBDE-100), 2,2',4,4',5,5'-hexabromodiphenyl ether (PBDE-153), 2,2',4,4',5,6'-hexabromodiphenyl ether (PBDE-154), 2,4,4'-tribromodiphenyl ether (PBDE-28), 2,2',4,4'-tetrabromodiphenyl ether (PBDE-47), 2,2',4,4',5-pentabromodiphenyl ether (PBDE-99), perfluorobutane sulfonic acid (PBFS), perfluorodecane sulfonic acid (PFDS), perfluorobutanoic acid (PFBA), perfluoroalkyl phosphonic acid (PFPA), perfluorohexane sulfonic acid (PFHxA), perfluorododecanoic acid (PFDoA), perfluorohexane sulfonic acid (PFHxS), perfluoroheptane sulfonic acid (PFHpS), perfluorooctane sulfonic acid (PFOS), perfluorooctane sulfonamide (PFOSA), N-Methyl perfluorooctane sulfonamidoacetic acid (N_MeFOSAA), N-Ethyl perfluorooctane sulfonamidoacetic acid (N_EtFOSAA), perfluoroheptanoic acid (PFHpA), perfluorooctanoic acid (PFOA), perfluorononanoic acid (PFNA), perfluorodecanoic acid (PFDA) and perfluoroundecanoic acid (PFUnA).

### Covariates

Logistic regression and BKMR models were adjusted for total plasma lipid content by the addition of a separate term in the model (ng/L, continuous), as recommended by Schisterman et al. [43]. The models were further adjusted for smoking status (never vs. ever), physical activity measured in metabolic equivalent tasks (MET)-hour/week (continuous), education (≤ 12 years, 12 to 14 years, > 14 years), personal history of benign breast disease (no vs. yes), family history of breast cancer (none, in first-degree relatives, in extended relatives), parity and age at first full-term pregnancy (FFTP) (no children, 1 or 2 children and < 30 years old at FFTP, ≥ 3 children and < 30 years old at FFTP, ≥ 30 years old at FFTP), total breastfeeding duration (never, ≤ 6 or > 6 months), age at menarche (years, continuous), current use of menopausal hormone therapy (yes, no), use of oral contraceptives (ever vs. never), age at menopause (menopause before age 51, menopause at age 51 or later). For the variables measured in different questionnaires, we took the value declared in the last questionnaire completed before the date of diagnosis of the case; for controls, the date of diagnosis of the matched case was used. Since BKMR and non-conditional logistic regression models do not account for case–control matching, these models were additionally adjusted for the matching criteria: age at blood draw (years, continuous), BMI at blood draw (kg/m^2^, continuous), and year of blood draw (continuous), except for menopausal status at blood draw, in order to avoid over-adjustment due to the a priori inclusion of age at menopause in the model.

The selection of confounders was done a priori, based on the known breast cancer risk factors available in the E3N dataset that are potentially associated with the exposures considered in the present study.

In our study population, missing values were < 5% for all variables and were imputed to the median (continuous variables) or modal value (categorical variables).

### Statistical analyses

Substances for which more than 25% of the values were below the limit of detection (LOD) were eliminated from the analysis (namely PBFS, PFDS, PFBA, PFPA, PFHxA, PFDoA). For those substances which had 25% or less of the values below the LOD, these last were imputed to ½ of the LOD.

Supplementary material Table 1 presents the percentage of values below the limit of detection for each substance measured in the present study. Finally, blood levels of 7 BFR (PBB-153, PBDE-100, 153, 154, 28, 47, 99) and 11 PFAS (PFHxS, PFHpS, PFOS, PFOSA, N_MeFOSAA, N_EtFOSAA, PFHpA, PFOA, PFNA, PFDA, PFUnA) were included in the models.

Demographic characteristics of the study participants were reported using means and standard deviations or counts with percentages. Univariate conditional logistic regression models were performed to compare concentrations of PFAS and BFR between cases and controls. Exposure to substances were log-transformed for the analyses. Correlations between log-transformed PFAS and BFR concentrations were assed using Pearson’s correlation tests.

#### Approach 1 – Principal Component Regression (PCR)

A PCA with varimax rotation was performed on the matrix of log-transformed biomarkers, to identify a reduced number of uncorrelated components representing the exposure to substances. The number of retained components was chosen using several indicators: individual and cumulated explained variance, and interpretability and coherence of identified components [40]. Then, multiple logistic regression models were fitted, with the identified components scores as main exposure variables (continuous and categorized in quintiles groups based on the adherence distribution to the different components). In order to verify if the same patterns of exposure were observed among cases and controls, PCA was also performed separately among these two groups.

In order to investigate the hypothesis of a differential relationship between exposure to PFAS and BFR and breast cancer risk based on the tumor expression of estrogen receptors (ER– vs. ER +), the components scores previously identified were also included in two separate models: the first model included ER + cases and all controls, while the second model included ER– cases and all controls. Cases for which information on the estrogen receptors’ expression was missing were excluded from the analysis.

All these models were adjusted for the covariates described above, including matching criteria.

As a sensitivity analysis, conditional logistic regression models accounting for the matching of cases and controls were run on the overall study population, but not on specific ER + and ER- subpopulations. These models were not adjusted for the matching criteria.

#### Approach 2 – Bayesian Kernel Machine Regression (BKMR)

BKMR was proposed as a new approach to assess the effect of exposure to chemical mixtures on health [34]. An R package (‘*bkmr*’) exists for this purpose, with the possibility of adapting the model to binary outcomes, like breast cancer [35]. In the present study, the hierarchical variable selection mode was used. There are two possible levels of variable selection: a group selection, corresponding to BFR on the one hand, and PFAS on the other hand, and an individual substance selection within each group.

The model for binary outcome using the Probit link function with hierarchical variable selection is as follows:

For each subject i = 1,…,n:

$${\Phi }^{-1}(\mathrm{P}({\mathrm{Y}}_{\mathrm{i}}=1)) =\mathrm{ h}[({\mathrm{Group}}_{1} = ({\mathrm{z}}_{\mathrm{i}1},\dots ,{\mathrm{z}}_{\mathrm{iM}}) , {\mathrm{Group}}_{2} = ({\mathrm{v}}_{\mathrm{i}1},\dots ,{\mathrm{v}}_{\mathrm{iN}})] +{\mathbf{x}}_{\mathrm{i}}^{\mathrm{T}}{\varvec{\upbeta}}+ {\upvarepsilon }_{\mathrm{i}}$$

where:

Φ^−1^ = probit link function;

Y_i_ = binary outcome (0/1), here breast cancer;

z_i1_, …,z_iM_, = exposure variables of group 1, here 11 PFAS (ng / mL of serum) as continuous, log-transformed and centered variables (i.e. subtraction of the mean);

v_i1_,…,v_iN_ = exposure variables of group 2, here 7 BFR (ng / L of plasma) as continuous, log-transformed and centered variables (i.e. subtraction of the mean);

h = flexible function of exposure variables, specified using a kernel function (exposure–response function);

**x**_**i**_ = vector of covariates (see the list below);

**β** = vector of the corresponding coefficients;

ε_i_ = residuals.

First, the BKMR model with a hierarchical selection of variables provides two types of posterior inclusion probabilities (PIPs): the PIPs of each of the two groups (BFR and PFAS), and the conditional PIPs of each substance within groups. The PIPs are indicators of the contribution of each group or substance in relation to the outcome.

Secondly, the BKMR method estimates a univariate exposure–response function for each substance. This function consists of a section of the function h quantifying the relationship between a given substance and the outcome, while all other exposure variables are set at their median value. In the specific case of a binary outcome, these sections of h can be interpreted as the relationship between the exposure variable and an underlying continuous latent outcome, such as a biomarker of health status underlying the binary outcome [35].

Then, the BKMR method provides bivariable exposure–response functions, which are estimates of the relationship between exposure to a given substance and the outcome while one other substance is fixed at predefined percentiles (20^th^, 50^th^, and 80^th^) and all the others are fixed at their median value. This approach allows to highlight interactions between pairs of substances: an alteration in the dose–response curve of one substance at a different percentile of another substance suggests an interaction, while parallel lines indicate no interaction. With the hierarchical variable selection mode, only interactions between substances not belonging to the same group can be identified.

Finally, the cumulative effect of the overall exposure to the substances is provided by calculating the differences between the estimated value of h when all substances are fixed at a predefined percentile, compared to the estimated value of h when all substances are fixed at the 50^th^ percentile, used as reference.

In the present study the number of iterations of the Markov Chain Monte Carlo sampler was set at 100,000 with non-informative default priors defined by the package.

As for approach 1, the model was performed first in the overall study population, and then separately for ER + cases and all controls, and for ER– cases and all controls, as described above.

As a sensitivity analysis, based on the results obtained from Pearson’s correlation tests and PCA, BKMR was also performed with a hierarchical selection including three groups: PFAS, PBDE, and, separately, PBB. All other parameters were kept identical to the main model.

The threshold for statistical significance was set at 5% and all statistical tests were two-sided. Statistical analyses were performed using SAS (version 9.4, SAS Institute) and R (version 4.0.3).

## Results

### Descriptive analyses

Table 1 shows summary statistics for each variable for the overall study population, as well as separately for cases and controls. Mean age at diagnosis was 68.5 years (range: 58.3–84.9 years). Among cases, the average follow-up time between blood sample collection and diagnosis was 12.2 years. Information on tumor estrogen receptor expression was available in 158 cases (81%) and among these 132 were ER + .

**Table 1:** Characteristics of the study population at the last questionnaire available before the date of diagnosis of cases (*N* = 388)

	**All (*N* = 388)**	**Breast cancer**		Global *p*-value*
		**Control (*N* = 194)**	**Case (*N* = 194)**	
Time of follow-up (years)	12.246 (1.947)	12.257 (1.955)	12.238 (1.944)	0.060
Education				0.580
≤ BAC	39 (10.052)	22 (11.340)	17 (8.762)	
BAC to BAC + 2	191 (49.226)	97 (50.000)	94 (48.454)	
> BAC + 2	158 (40.722)	75 (38.660)	83 (42.784)	
Physical activity (metabolic equivalent tasks (MET)-hour/week)	44.513 (36.840)	46.134 (37.878)	42.892 (35.797)	0.388
Smoking status				0.184
Never	187 (48.196)	87 (44.845)	100 (51.546)	
Ever	201 (51.804)	107 (55.155)	94 (48.454)	
Body masse index at blood draw (kg/m2)	23.815 (3.553)	23.850 (3.614)	23.779 (3.500)	0.763
Family history of breast cancer				0.283
None	278 (71.650)	144 (74.227)	134 (69.072)	
In first-degree relatives	59 (15.206)	30 (15.464)	29 (14.949)	
In extended relatives	51 (13.144)	20 (10.309)	31 (15.979)	
Personal history of benign breast disease				0.111
No	224 (57.732)	120 (61.856)	104 (53.608)	
Yes	164 (42.268)	74 (38.144)	90 (46.392)	
Age at menarche (year)	12.807 (1.378)	12.745 (1.446)	12.869 (1.306)	0.370
Age at menopause				0.437
Menopause before age 51	189 (48.711)	98 (50.515)	91 (46.907)	
Menopause at age 51 or later	199 (51.289)	96 (49.485)	103 (53.093)	
Use of oral contraceptives				0.420
Never	133 (34.278)	70 (36.082)	63 (32.474)	
Ever	255 (65.722)	124 (63.918)	131 (67.526)	
Current use of menopausal hormone therapy				0.235
No	301 (77.577)	155 (79.897)	146 (75.258)	
Yes	87 (22.423)	39 (20.103)	48 (24.742)	
Parity and age at first full-term pregnancy (FFTP)				0.171
No children	55 (14.174)	22 (11.340)	33 (17.010)	
1 or 2 children and < 25 years old at FFTP	192 (49.485)	95 (48.969)	97 (50.000)	
≥ 3 children and < 25 years old at FFTP	106 (27.320)	61 (31.443)	45 (23.196)	
≥ 25 years old at FFTP	35 (9.021)	16 (8.248)	19 (9.794)	
Total breastfeeding duration in months				0.343
Never	150 (38.659)	79 (40.722)	71 (36.598)	
≤ 6	162 (41.753)	83 (42.784)	79 (40.722)	
> 6	76 (19.588)	32 (16.494)	44 (22.680)	
Adherence score to Prudent dietary pattern				0.053
< median	193 (49.742)	87 (44.845)	106 (54.639)	
≥ median	195 (50.258)	107 (55.155)	88 (45.361)	
Adherence score to Western dietary pattern				0.915
< median	193 (49.742)	96 (49.485)	97 (50.000)	
≥ median	195 (50.258)	98 (50.515)	97 (50.000)	

Mean (Standart deviation) and Counts (Percentages) are presented for continuous and categorical variables respectively

Among BFR, PBDE-47 (4.70 ng/L) was the congener with the highest average concentration in the overall population, followed by PBDE-153 (3.14 ng/L) and PBB-153 (2.03 ng/L). PFOS (19.08 ng/mL) and PFOA (7.35 ng/mL) were the two PFAS with the highest average concentration in the study population. The mean and standard deviation of each exposure variable in the total study population, and for cases and controls separately, is presented in Table 2. Overall, the results of Pearson’s rank correlation tests, reported in Supplementary material Fig. 1, highlighted how all PBDE were positively and strongly correlated, while no or weak correlations were observed between PBDE and PBB-153. Also PFAS were generally positively correlated, while between PFAS and PBDE, as well as between PFAS and PBB-153, no or weak correlations were observed.

**Table 2:** Internal BFR and PFAS exposure levels of the study population (*n* = 388)

		Breast cancer status		
	All (*N* = 388)	Control (*N* = 194)	Case (*N* = 194)	*p*-value^a^
PBB-153 in ng/L of plasma	2.036 (3.042)	1.959 (1.326)	2.114 (4.098)	0.6267
PBDE-100 in ng/L of plasma	1.302 (1.573)	1.354 (1.811)	1.251 (1.295)	0.5254
PBDE-153 in ng/L of plasma	3.144 (1.520)	3.122 (1.549)	3.167 (1.494)	0.7772
PBDE-154 in ng/L of plasma	0.205 (0.248)	0.217 (0.318)	0.194 (0.148)	0.3696
PBDE-28 in ng/L of plasma	0.329 (0.411)	0.326 (0.415)	0.332 (0.407)	0.8872
PBDE-47 in ng/L of plasma	4.702 (7.136)	4.758 (7.577)	4.646 (6.685)	0.8768
PBDE-99 in ng/L of plasma	1.203 (2.817)	1.276 (3.652)	1.129 (1.603)	0.6172
N-EtFOSAA in ng/mL of serum	0.903 (1.119)	0.949 (1.373)	0.856 (0.788)	0.4280
N-MeFOSAA in ng/mL of serum	0.745 (1.041)	0.745 (1.267)	0.745 (0.753)	0.9960
PFDA in ng/mL of serum	0.303 (0.143)	0.310 (0.164)	0.295 (0.117)	0.2906
PFHpA in ng/mL of serum	0.275 (0.266)	0.272 (0.292)	0.279 (0.238)	0.7734
PFHxS in ng/mL of serum	2.126 (1.602)	2.129 (1.514)	2.122 (1.690)	0.9663
PFNA in ng/mL of serum	0.757 (0.401)	0.777 (0.486)	0.738 (0.292)	0.3414
PFOA in ng/mL of serum	7.345 (3.510)	7.279 (3.259)	7.410 (3.752)	0.6822
PFOS in ng/mL of serum	19.077 (8.228)	18.745 (7.782)	19.410 (8.658)	0.4128
PFOSA in ng/mL of serum	0.857 (0.480)	0.840 (0.462)	0.875 (0.498)	0.4330
PFUnA in ng/mL of serum	0.211 (0.098)	0.214 (0.101)	0.207 (0.096)	0.4531
PFHpS in ng/mL of serum	0.443 (0.186)	0.440 (0.177)	0.446 (0.194)	0.7577

Mean (Standart deviation) are presented

^a^Wald test *p*-value from univariate conditional logistic regressions

### Approach 1 – Principal Component Regression

PCA identified four main components accounting respectively for the 29%, 22%, 9%, and 8% of the total variance. Loading factors for each chemical on each component are presented in Supplementary material Table 2. The first Component was characterized by high loading factors for all PBDE. The second Component had high loading factors for all PFAS except PFOSA, N_MeFOSAA, N_EtFOSAA and PFHpA. The third Component had high loading factors for PFOSA, N_MeFOSAA and N_EtFOSAA. Finally, the fourth Component had high loading factors for PBDE-153, PBB-153, PFDA and PFUnA. Similar patterns were observed when running PCA separately among cases and controls (data not shown).

Results for logistic regression models are presented in Table 3. When including all breast cancer cases, a positive association between Component 3 in quintiles and breast cancer risk was observed, with Odds Ratios (OR) > 1 for all quintile groups when compared to the first quintile group (global p-value = 0.09).

**Table 3:** Associations between adherence to PCA components and all breast cancer, ER + breast cancer and ER- breast cancer risk. Components are used in continuous and in quintiles in logistic regression models. Odds Ratio (OR) and 95% Confidence Intervals (CI) are presented

	**All breast cancer**				**ER + breast cancer**				**ER- breast cancer**			
	Number (%)—Control	Number (%)—Cases	OR [95% CI]	*p*-value	Number (%)—Control	Number (%)—Cases	OR [95% CI]	*p*-value	Number (%)—Control	Number (%)—Cases	OR [95% CI]	*p*-value
	*N* = 194	*N* = 194			*N* = 194	*N* = 132			*N* = 194	*N* = 26		
Component 1, continuous	194 (100.00)	194 (100.00)	1.01 [0.82; 1.25]	0.9208	194 (100.00)	132 (100.00)	1.03 [0.81; 1.31]	0.8068	194 (100.00)	26 (100.00)	0.74 [0.41; 1.33]	0.3169
Component 1, quintiles				0.2800				0.5932				0.0566
Quintile 1	45 (23.20)	32 (16.49)	Reference		45 (23.20)	26 (19.70)	Reference		45 (23.20)	3 (11.54)	Reference	
Quintile 2	33 (17.01)	45 (23.20)	**2.24 [1.09; 4.60]**		33 (17.01)	29 (21.97)	1.55 [0.70; 3.44]		33 (17.01)	7 (26.92)	5.90 [0.83; 41.99]	
Quintile 3	40 (20.62)	38 (19.59)	1.55 [0.75; 3.20]		40 (20.62)	22 (16.67)	1.12 [0.50; 2.52]		40 (20.62)	8 (30.77)	4.57 [0.71; 29.61]	
Quintile 4	40 (20.62)	38 (19.59)	1.38 [0.67; 2.84]		40 (20.62)	22 (16.67)	1.05 [0.46; 2.35]		40 (20.62)	7 (26.92)	3.43 [0.55; 21.20]	
Quintile 5	36 (18.56)	41 (21.13)	1.65 [0.80; 3.40]		36 (18.56)	33 (25.00)	1.86 [0.86; 4.03]		36 (18.56)	1 (3.85)	0.11 [0.01; 2.31]	
Component 2, continuous	194 (100.00)	194 (100.00)	0.97 [0.78; 1.21]	0.8043	194 (100.00)	132 (100.00)	1.04 [0.81; 1.34]	0.7578	194 (100.00)	26 (100.00)	1.31 [0.77; 2.23]	0.3136
Component 2, quintiles				0.6753				0.7218				0.3073
Quintile 1	38 (19.59)	39 (20.10)	Reference		38 (19.59)	24 (18.18)	Reference		38 (19.59)	1 (3.85)	Reference	
Quintile 2	43 (22.16)	35 (18.04)	0.76 [0.38; 1.54]		43 (22.16)	22 (16.67)	0.80 [0.35; 1.81]		43 (22.16)	7 (26.92)	10.46 [0.85; 128.74]	
Quintile 3	35 (18.04)	43 (22.16)	0.93 [0.46; 1.89]		35 (18.04)	31 (23.48)	1.13 [0.51; 2.51]		35 (18.04)	6 (23.08)	4.45 [0.37; 53.96]	
Quintile 4	37 (19.07)	41 (21.13)	1.07 [0.53; 2.17]		37 (19.07)	29 (21.97)	1.22 [0.55; 2.72]		37 (19.07)	7 (26.92)	8.31 [0.69;100.26]	
Quintile 5	41 (21.13)	36 (18.56)	0.66 [0.31; 1.39]		41 (21.13)	26 (19.70)	0.91 [0.39; 2.13]		41 (21.13)	5 (19.23)	3.24 [0.22; 46.87]	
Component 3, continuous	194 (100.00)	194 (100.00)	1.09 [0.88; 1.36]	0.4141	194 (100.00)	132 (100.00)	1.19 [0.93; 1.51]	0.1702	194 (100.00)	26 (100.00)	1.20 [0.76; 1.88]	0.4374
Component 3, quintiles				0.0882				**0.0397**				0.5452
Quintile 1	45 (23.20)	32 (16.49)	Reference		45 (23.20)	18 (13.64)	Reference		45 (23.20)	6 (23.08)	Reference	
Quintile 2	39 (20.10)	39 (20.10)	1.33 [0.66; 2.66]		39 (20.10)	23 (17.42)	1.40 [0.61; 3.24]		39 (20.10)	4 (15.38)	1.03 [0.18; 6.04]	
Quintile 3	37 (19.07)	41 (21.13)	1.72 [0.86; 3.48]		37 (19.07)	33 (25.00)	**2.61 [1.16; 5.88]**		37 (19.07)	4 (15.38)	1.88 [0.34; 10.58]	
Quintile 4	29 (14.95)	49 (25.26)	**2.47 [1.21; 5.06]**		29 (14.95)	34 (25.76)	**3.06 [1.36; 6.89]**		29 (14.95)	7 (26.92)	3.18 [0.66; 15.45]	
Quintile 5	44 (22.68)	33 (17.01)	1.09 [0.54; 2.21]		44 (22.68)	24 (18.18)	1.59 [0.70; 3.61]		44 (22.68)	5 (19.23)	0.97 [0.18; 5.18]	
Component 4, continuous	194 (100.00)	194 (100.00)	0.93 [0.74; 1.18]	0.5660	194 (100.00)	132 (100.00)	0.91 [0.70; 1.19]	0.4861	194 (100.00)	26 (100.00)	0.71 [0.41; 1.23]	0.2170
Component 4, quintiles				0.1077				0.0531				0.6901
Quintile 1	39 (20.10)	38 (19.59)	Reference		39 (20.10)	25 (18.94)	Reference		39 (20.10)	6 (23.08)	Reference	
Quintile 2	39 (20.10)	39 (20.10)	1.26 [0.62; 2.53]		39 (20.10)	26 (19.70)	1.15 [0.51; 2.56]		39 (20.10)	6 (23.08)	1.41 [0.27; 7.52]	
Quintile 3	30 (15.46)	48 (24.74)	1.94 [0.93; 4.03]		30 (15.46)	37 (28.03)	2.14 [0.94; 4.89]		30 (15.46)	6 (23.08)	1.37 [0.23; 8.13]	
Quintile 4	43 (22.16)	35 (18.04)	0.89 [0.43; 1.83]		43 (22.16)	22 (16.67)	0.80 [0.34; 1.85]		43 (22.16)	5 (19.23)	0.65 [0.10; 4.12]	
Quintile 5	43 (22.16)	34 (17.53)	0.79 [0.37; 1.65]		43 (22.16)	22 (16.67)	0.69 [0.29; 1.66]		43 (22.16)	3 (11.54)	0.45 [0.07; 2.79]	

All models are adjusted on age at blood draw (continuous), body mass index at blood draw (kg/m^2^, continuous), year of blood draw (continuous), total plasma lipid content (ng/L, continuous), smoking status (never vs. ever), physical activity measured in metabolic equivalent tasks (MET)-hour/week (continuous), education (≤ 12 years, 12 to 14 years, > 14 years), personal history of benign breast disease (no vs. yes), and family history of breast cancer (none, in first-degree relatives, in extended relatives), parity and age at first fullterm pregnancy (FFTP) (no children, 1 or 2 children and < 30 years old at FFTP, ≥ 3 children and < 30 years old at FFTP, ≥ 30 years old at FFTP), total breastfeeding duration (never, ≤ 6 or > 6 months), age at menarche years, continuous), current use of menopausal hormone therapy (yes, no), use of oral contraceptives (ever vs. never), menopausal status and age at menopause (menopause before age 51, menopause at age 51 or later)

Results were similar when including only ER + cases: Component 3 in quintiles was positively associated with ER + breast cancer risk, with OR > 1 for all quintile groups when compared to the first quintile group (global *p*-value = 0.04). Also, an association was highlighted for Component 4, with OR > 1 for the second and third quintile groups, and OR < 1 for the fourth and fifth quintile groups (global *p*-value = 0.05).

Finally, when including only ER- cases, an association was identified for Component 1 in quintiles, with OR > 1 for the second, third, and fourth quintile groups, and OR < 1 for the fifth quintile group (global *p*-value = 0.06). In addition, it has to be taken into account that all the confidence intervals were wide for this analysis due to the limited number of cases included (*n* = 26).

### Sensitivity analysis

When performing conditional logistic regression models including the overall population, results were similar to those obtained with unconditional logistic regression models in the overall population (Supplementary material Table 3).

### Approach 2 – Bayesian Kernel Machine Regression (BKMR)

The PIPs of BFR and PFAS groups were 0.43 and 0.57, respectively. Among BFR, the substances that had the highest conditional PIPs were PBDE-153 (0.21), and PBB-153 (0.18), while among PFAS, PFOSA (0.16) was the substance with the highest conditional PIP (Supplementary material Fig. 2-A).

**Fig. 1:**
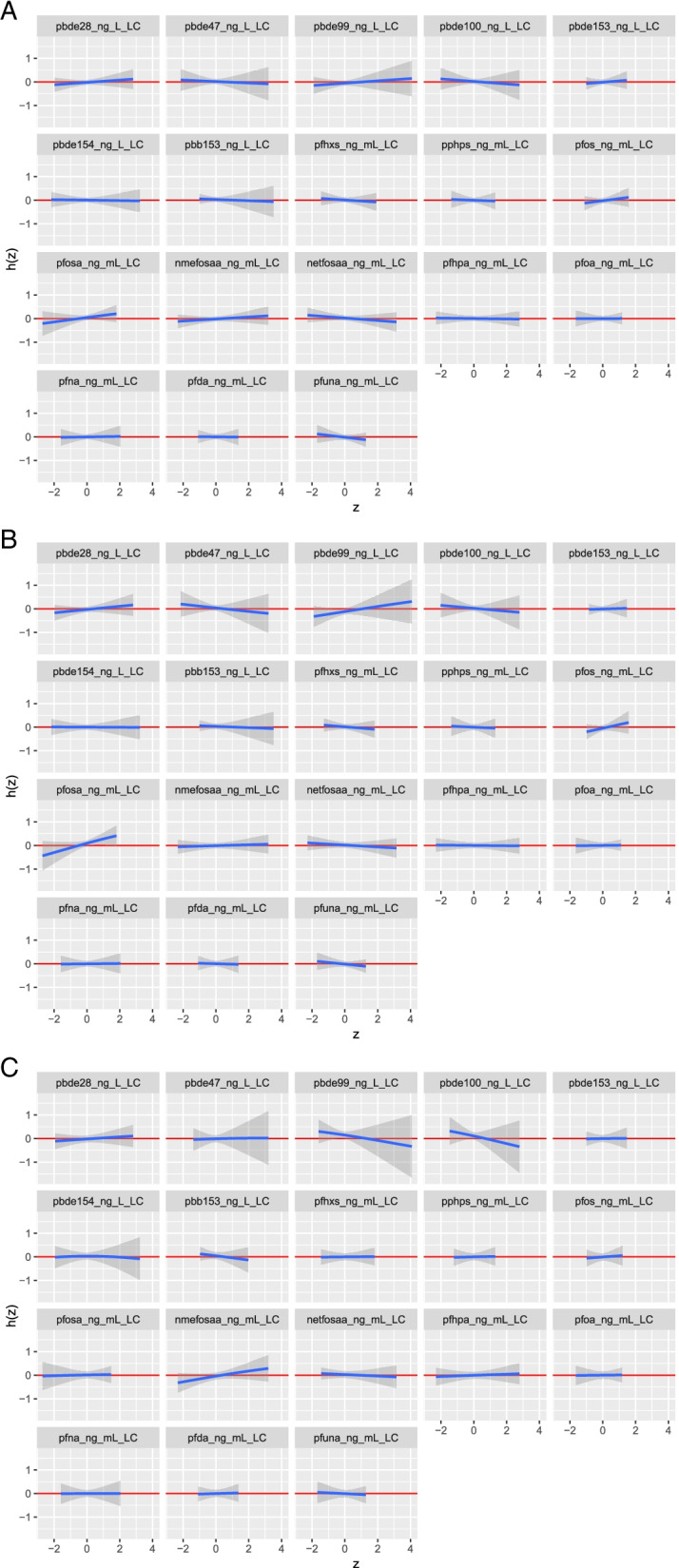
Univariate exposure–response functions between exposure to each substance and probit of probability of: **A**: having a breast cancer, **B:** having an ER + breast cancer, **C:** having an ER- breast cancer, all other substances being fixed at their median value

Regarding the relationship between each substance and breast cancer risk while setting all other exposure variables at their median value, concerning the BFR, a positive trend was observed with PBDE-28, and PBDE-99, while an inverse relationship was observed with PBDE-47 and PBDE-100. Concerning PFAS, a positive trend was observed for PFOS, PFOSA, and N_MeFOSAA, while a negative trend was observed for N_EtFOSAA, and PFUnA (Fig. 1-A).

Regarding the interactions plots, no interactions were identified between substances across groups (Supplementary material Fig. 3).

**Fig. 2:**
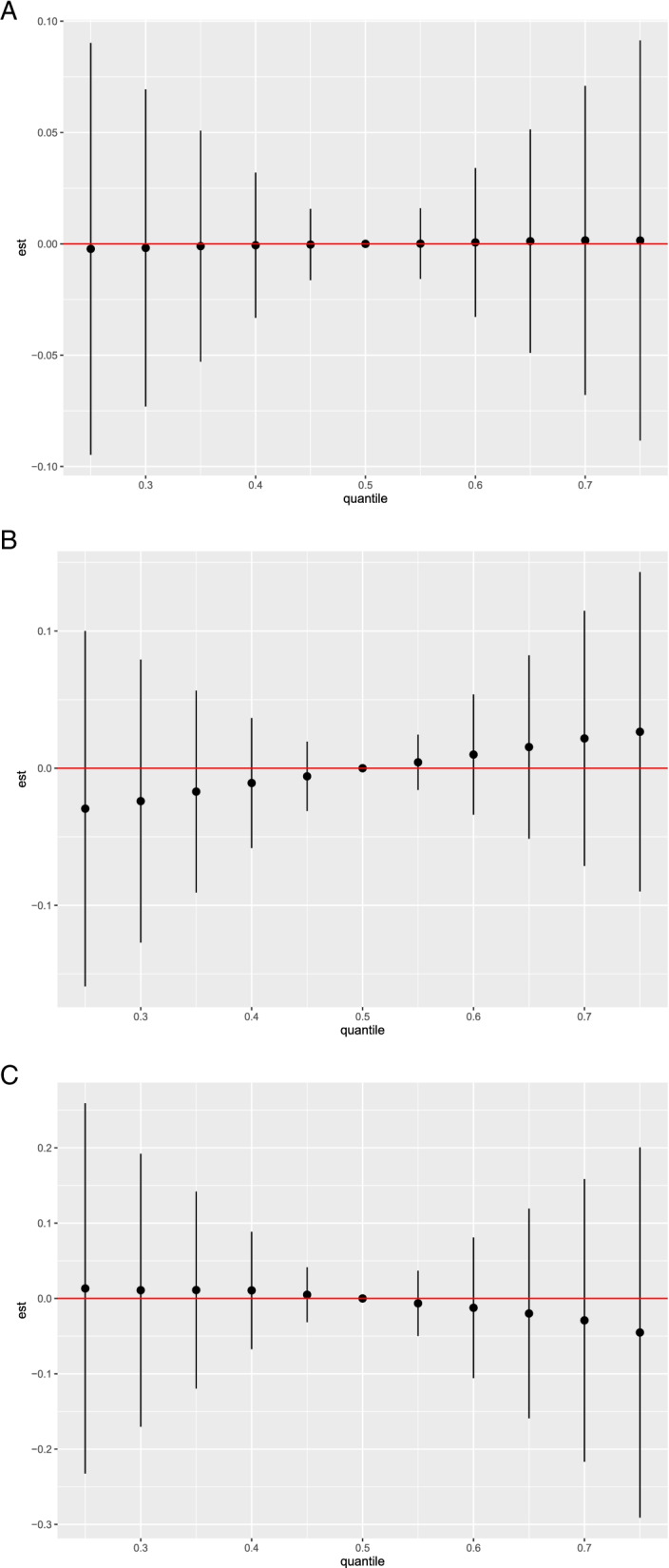
Cumulative effect of PFAS and BFR for: **A**: All breast cancer risk. **B:** ER + breast cancer risk. **C:** ER- breast cancer risk

Finally, the cumulative effect of PFAS and BFR, meaning the cumulative effect estimate comparing all substances at their median concentrations (reference) to the concentrations corresponding to each 5th percentile from the 25th to the 75th percentile, was close to zero (Fig. 2-A).

When performing the analyses including only ER + cases, the PIPs of BFR and PFAS were respectively 0.48 and 0.66. The conditional PIPs of each of the substances within BFR were similar to those observed when including all breast cancer cases, while for PFAS the substances for which a higher conditional PIP was observed were PFOSA (0.34) and PFOS (0.15) (Supplementary material Fig. 2-B). The univariate exposure–response functions showed similar results as for the overall population, but with steeper slopes, especially for PBDE-47, PBDE-99 and PFOSA (Fig. 1-B). No interactions were identified between substances across groups (data not shown). A slightly positive association was observed between PFAS and BFR cumulative effect and ER + breast cancer risk (Fig. 2-B).

The PIPs of BFR and PFAS, when including only ER- breast cancer, were respectively 0.74 and 0.62. The highest conditional PIPs were observed for PBDE-100 (0.25) and PBDE-99 (0.18) concerning BFR, while with regards to PFAS the substance with the highest conditional PIP was N_MeFOSAA (0.23) (Supplementary material Fig. 2-C). The results obtained from the univariate exposure–response functions, highlighted an inverse association between PBDE-99 as well as PBDE-100 and ER- breast cancer risk, and a positive association, between N_MeFOSAA and ER- breast cancer risk. (Fig. 1-C). No interactions were identified between substances across groups (data not shown). Finally, the cumulative effect of PFAS and BFR was associated with a decrease of ER- breast cancer risk (Fig. 2-C).

### Sensitivity analysis

When running BKMR including PBDE and PBB as separate groups, the PIPs were 0.38, 0.33, and 0.50 for PBDE, PBB, and PFAS, respectively. The conditional PIPs were similar to those obtained in the main analysis, expect for PBB-153 which, being the only substance included in the PBB group, had a conditional PIP equal to 1.00. The results of the univariate exposure–response functions were comparable to those obtained in the main analysis, although the associations were overall attenuated (data not shown). No interactions were identified between substances across groups (data not shown). The cumulative effect of PFAS, PBDE, and PBB on the risk of breast cancer was close to zero (Supplementary material Fig. 4).

## Discussion

In this study, we explored the association between exposure to BFR and PFAS as a mixture and breast cancer risk, comparing two statistical approaches, PCR and BKMR. Globally, no clear association between mixtures of BFR and PFAS circulating levels with breast cancer risk was highlighted and no interaction between substances was identified. However, both approaches suggested a differential effect on ER + and ER- breast cancer risk, although the results for ER- breast cancer should be interpreted carefully due to the small number of ER- cases included in the study.

Regarding results obtained with PCR, an association between Component 3 (mainly composed of PFOSA, N_MeFOSAA and N_EtFOSAA) and ER + breast cancer risk was identified. A non-linear association could be noticed for Component 4 (mainly composed of PBDE 153, PBB 153, PFDA and PFUnA). As expected, results for overall breast cancer risk were similar to those obtained for ER + since these represented the majority (81%) of breast cancer cases included in the study.

In the ER- stratum, a non-linear association could be noticed for Component 1 (mainly composed of PBDEs). The small number of ER- cases could explain the lack of statistically significant effect.

When performing BKMR analyses for overall and ER + breast cancer risk, results were also very similar, but globally attenuated for overall breast cancer risk. The PFAS group had a slightly higher PIP than the BFR group, suggesting that PFAS could play a more important role than BFR in the occurrence of breast cancer. Moreover, conditional PIPs highlighted that PBDE-153 (positive relationship) and PBB-153 (negative relationship), among the BFR group, and PFOSA (positive relationship), among the PFAS group, played the most important roles in the relationship with overall and ER + breast cancer risk. A positive cumulative effect of BFR and PFAS on ER + breast cancer risk was also highlighted. No effect was seen for overall breast cancer risk, probably due to a dilution effect due to the inclusion of ER- cases.

When performing analyses including only ER- breast cancer cases, PIPs were instead slightly higher for the BFR group than the PFAS group. In addition, the substances that had the highest PIPs were PBDE-100 and PBDE-99, in the BFR group, and N_MeFOSAA, in the PFAS group. Regarding the relationship between each substance and ER- breast cancer risk, several differences were noticed in comparison to ER + breast cancer risk. Indeed, PBDE-99 and ER- breast cancer risk showed a negative relationship; stiffer slopes were identified for PBDE-100 (negative relationship) and for N_MeFOSAA (positive relationship) compared to ER + breast cancer risk, while the slopes for PFOSA and PFOS were milder. Finally, the results suggest a negative cumulative effect of BFR and PFAS on ER- breast cancer risk.

When comparing the results obtained with the two approaches tested in the present study, some consistency can be noticed. Indeed, with regard to ER + breast cancer risk, an association was shown with Component 3, and a non-linear association for Component 4. This was overall coherent with the results of the BKMR approach, which identified among the most important substances contributing to ER + breast cancer risk, PBDE-153 and PBB-153, with a positive and a negative relationship, respectively. These two substances were in fact contributing strongly to Component 4, and this could explain the non-linear association identified for this Component. Concerning the PFAS group, the BKMR approach identified PFOSA among the substances with higher PIP, highlighting a positive relationship with ER + breast cancer risk. PFOSA, in turn, was also an important contributor to Component 3 and could be responsible for the association identified between this component and ER + breast cancer risk.

With regard to ER- breast cancer risk, only a non-linear association was highlighted for Component 1. Among the substances with the higher PIPs identified by the BKMR approach, some were contributing to Component 1, such as PBDE-99 and PBDE-100, having both a negative relationship with ER- breast cancer risk. However, numerous other substances contributed to Component 1, with opposite relationships with ER- breast cancer risks and this could explain the non-linear association found with this Component.

PCA, a dimension reduction method classically used in epidemiology to deal with multicollinearity, transforms a large set of variables into a smaller one, minimizing the loss of information. It does so by creating new uncorrelated variables, called components, on the basis of correlations between the initial variables [40]. In our study, PCA has identified components that were linear combination of initial exposures, and logistic regression models further allowed to identify some associations between these components and breast cancer risk. Thereby this approach does not allow concluding whether all the substances contributing to the component, or rather only some of them, are responsible for the relationship with the outcome. Given that components are linear combinations of substances, this approach does not allow either to identify interactions between substances.

The BKMR approach allows to model non-linear and non-additive relationships between substances and outcome, accounting for confounding variables [34]. For hierarchical variable selection, suitable for studying multiple correlated substances, groups of substances are built a priori, based on the correlations between substances but also on the known potential biological mechanisms [24, 35]. In this study, we only tested two and three groups, separating the main families of substances known to have similar chemical structure and industrial use. Finer groupings of substances according to different biological mechanisms could be considered in future studies, thus testing other hypotheses. In our study, results obtained with BKMR models allowed to generate hypothesis concerning which substances play the most important role in the relationship with breast cancer risk by means of the PIPs and the shape of the slopes. While this method allows identifying interactions between substances across groups, no interaction has been identified in the present study, possibly due to the lack of statistical power.

### Comparison of results with other studies

Previous studies have evaluated the associations between internal exposure levels of BFR and PFAS and breast cancer [18–29]. These studies focused on exposures to individual substances, or exposures to sums of substances that are often highly correlated. The results obtained by these studies are contradictory, with positive, negative, or no associations identified with the individual substances or sums of substances studied [18–29].

More precisely, regarding the hypothesis of a positive association between PFOSA and both ER + and all breast cancer risk generated by our study, one previous case–control study has also identified a positive relationship between PFOSA and all breast cancer risk [21], while another one has identified no association for all breast cancer risk or ER + breast cancer risk [20]. Concerning the hypothesis of a positive association between PBDE-153 and both ER + and all breast cancer risk generated by our study, one previous case–control study has also identified a positive relationship between PBDE-153 and all breast cancer risk but not ER + breast cancer risk [27], while others studies have identified no associations [24, 26]. Previous analyses in the same E3N nested case–control population using a single-pollutant approach have identified a negative association for both all breast cancer risk and ER + breast cancer risk [23]. Finally, regarding the hypothesis of a negative association between PBB-153 and both ER + and all breast cancer risk, previous analyses in the same E3N nested case–control population have identified no association for all breast cancer risk or ER + breast cancer risk [23].

However, comparisons between the results of these studies, using a single pollutant approach, and our results, obtained from a mixture approach, must be made with caution. Indeed, the high correlation levels between substances could potentially lead to biased estimates of the associations between individual substances and breast cancer and this could explain the inconstant results between the different studies. Moreover, although the use of sums of substance, instead of individual substances, could have overcome the problems due to collinearity, this approach is based on the strong assumption that the aggregated substances have an additive effect, which is not necessarily true. The limits of these methods and the lack of studies evaluating mixtures of substances rather than individual or sums of substances could explain the global inconsistency of results. More studies assessing the impact of the choice between multi-pollutants versus single-pollutant approaches on results are needed to better understand these differences.

### Strengths and weaknesses

This study presents some limitations. First, the study population was composed of women working for the French national education system, with globally a healthier lifestyle than the general population, so the extrapolation of results to the general population should be done with caution. In addition, the small study population size limited the statistical power. In particular, the small number of ER- cases limited the consistency of the results when performing stratified analysis based on hormone receptor status. The presence of chemicals with observations below the limit of detection should also be taken into account. We acknowledge that large left-censored data (i.e. > 25%) may have impacted regression based estimates, especially when using parametric methods constrained by distributional assumptions, which is not the case of BKMR [44]. To overcome this issue, we excluded substances for which more than the 25% of the values were left-censored; for such substances we opted for a substitution method (by LOD/2) considering the computational convenience and the little impact of the method chosen for managing low percentages of non-detects (< 25%) in principal component analysis [45]. Furthermore, the long average time between measurements and diagnosis in cases could have limited the ability to identify significant effects. Moreover, as mentioned earlier, when applying BKMR models, chemicals were grouped based on similar chemical structure and industrial use, thus limiting the hypothesis tested. Additionally, the single-measurement of exposures did not allow taking into account the trajectories of exposures to these substances over time. Finally, the current BKMR method available for binary outcomes is implemented with a probit link function instead of logit, which is more commonly used in case–control studies. However, we believe that the findings from our exploratory approach are not affected by the underlying link function because of their mathematical similarity whose differences would mainly affect testing or interpretation of the results [46, 47]. In addition, we tried to take into account the matched design by adding the matching variable in the models.

The present study has also several strengths. First, the availability of numerous information collected in the E3N cohort has permitted to adjust models on main potential confounders for breast cancer. In addition, the present study investigated two methodological approaches, PCR and BKMR, that are adapted to the assessment of the effects of chemical mixtures rather than the single-exposures, and that take into account multicollinearity. These features allowed to highlight some positive trends between some PBDEs and PFAS considered as mixtures and breast cancer risk, which could be elusive in single-pollutant models. In addition, the combination of these two methods allowed generating more robust conclusions than those that could have been obtained applying a single method. In addition, these two approaches can be seen as complementary, PCR detecting associations with some components of correlated substances, and BKMR further allowing to identify interactions between substances and non-linear associations while accommodating confounding.

## Conclusions

Combining the results of these two approaches has made it possible to formulate hypotheses about the components associated with breast cancer risk and to further hypothesize which substances contributing to these components could be responsible for the association identified, highlighting the direction of the relationship and taking multicollinearity into account. However, the insufficient number of subjects has not allowed identifying some potentially significant association with the BKMR approach, which resulted in very large credible intervals. Further studies evaluating mixtures of substances on larger sample sizes are needed.

## Supplementary Information

**Additional file 1: Table S1.** Percentage of values below the Limit Of Detection (LOD) and associated decision for each substance measured in the present study. **Table S2**. Loading factors for each substances on each of the 4 components obtained by principal component analysis. **Table S3**. Associations between adherence to PCA components and all breast cancer risk. Components are used in continuous and in quintiles in conditionnal logistic regression models. Odds Ratio (OR) and 95% Confidence Intervals (CI) are presented. **Figure S1.** Correlation matrix between log-transformed exposure to substances in the study population. Pearson’s rank correlation coefficients are presented. **Figure S2-A**. Conditional posterior inclusion probabilities of substances within group for all breast cancer risk. **Figure S2-B.** Conditional posterior inclusion probabilities of substances within group for ER+ breast cancer risk. **Figure S2-C**. Conditional posterior inclusion probabilities of substances within group for ER- breast cancer risk. **Figure S3**. Exposure-response functions between exposure to Substance 1 and probit of probability of having a breast cancer while Substance 2 is fixed at defined percentiles (20th, 50th, and 80th), all other substances being fixed at their median value. **Figure S4**. Cumulative effect of PFAS, PBB and PBDE for all breast cancer risk.

## Data Availability

The datasets used and/or analysed during the current study are available from the corresponding author on reasonable request.

## Abbreviations

BFR: Brominated flame retardants
BKMR: Bayesian Kernel Machine Regression
BMI: Body mass index
CI: Confidence Interval
EDCs: Endocrine disrupting chemicals
ER +: Estrogen Receptor positive
ER-: Estrogen Receptor negative
FFTP: First full-term pregnancy
IARC: International Agency for Research on Cancer
LOD: Limit of detection
MET: Metabolic equivalent tasks
MGEN: Mutuelle Générale de l'Education Nationale
OR: Odds Ratio
PBB: Polybrominated biphenyls
PBB-153: 2,2',4,4',5,5'-Hexabromobiphenyl
PBDE: Polybrominated diphenyl ethers
PBDE-28: 2,4,4'-Tribromodiphenyl ether
PBDE-47: 2,2',4,4'-Tetrabromodiphenyl ether
PBDE-99: 2,2',4,4',5-Pentabromodiphenyl ether
PBDE-100: 2,2',4,4',6-Pentabromodiphenyl ether
PBDE-153: 2,2',4,4',5,5'-Hexabromodiphenyl ether
PBDE-154: 2,2',4,4',5,6'-Hexabromodiphenyl ether
PCA: Principal Component Analysis
PCR: Principal Component Regression
PFAS: Per- and polyfluorinated alkylated substances
PFBS: Perfluorobutane sulfonic acid
PFDA: Perfluorodecanoic acid
PFBA: Perfluorobutanoic acid
PFDoA: Perfluorododecanoic acid
PFDS: Perfluorodecane sulfonic acid
PFHpA: Perfluoroheptanoic acid
PFHxA: Perfluorohexane sulfonic acid
PFHpS: Perfluoroheptane sulfonic acid
PFHxS: Perfluorohexane sulfonic acid
PFNA: Perfluorononanoic acid
PFOA: Perfluorooctanoic acid
PFOS: Perfluorooctane sulfonic acid
PFOSA: Perfluorooctane sulfonamide
PFPA: Perfluoroalkyl phosphonic acid
PFUnA: Perfluoroundecanoic acid
PIP: Posterior inclusion probabilities
N_EtFOSAA: N-Ethyl perfluorooctane sulfonamide
N_MeFOSAA: N-Methyl perfluorooctane Sulfonamide
