# Supplementary material for: Application of two statistical approaches (Bayesian Kernel Machine Regression and Principal Component Regression) to assess breast cancer risk in association to exposure to mixtures of brominated flame retardants and per- and polyfluorinated alkylated substances in the E3N cohort
Source: Environ Health. 2022 Feb 26;21:27. doi: 10.1186/s12940-022-00840-4 (PMC8881807; doi:10.1186/s12940-022-00840-4)

**Supplementary materials**

[Supplementary table 1: Percentage of values below the limit of detection (LOD) and associated decision for each substance measured in the present study. 2](#_Toc93058534)

[Supplementary table 2: Loading factors for each substances on each of the 4 components obtained by principal component analysis 3](#_Toc93058535)

[Supplementary table 3: Associations between adherence to PCA components and all breast cancer risk. Components are used in continuous and in quintiles in conditionnal logistic regression models. Odds Ratio (OR) and 95% Confidence Intervals (CI) are presented. 4](#_Toc93058536)

[Supplementary figure 1: Correlation matrix between log-transformed exposure to substances in the study population. Pearson’s rank correlation coefficients are presented. 5](#_Toc93058537)

[Supplementary figure 2-A: Conditional posterior inclusion probabilities of substances within group for all breast cancer risk. 6](#_Toc93058538)

[Supplementary figure 2-B: Conditional posterior inclusion probabilities of substances within group for ER+ breast cancer risk. 7](#_Toc93058539)

[Supplementary figure 2-C: Conditional posterior inclusion probabilities of substances within group for ER- breast cancer risk. 7](#_Toc93058540)

[Supplementary figure 3: Exposure-response functions between exposure to Substance 1 and probit of probability of having a breast cancer while Substance 2 is fixed at defined percentiles (20th, 50th, and 80th), all other substances being fixed at their median value. 8](#_Toc93058541)

[Supplementary figure 4: Cumulative effect of PFAS, PBB and PBDE for all breast cancer risk. 12](#_Toc93058542)

# Supplementary table 1: Percentage of values below the limit of detection (LOD) and associated decision for each substance measured in the present study.

|  | Substance | % of values <LOD | Decision |
| --- | --- | --- | --- |
| BFR in ng/L of serum | PBDE-28 in ng/L of plasma | 1% | Imputation to ½ LOD |
|  | PBDE-47 in ng/L of plasma | 0% |  |
|  | PBDE-99 in ng/L of plasma | 0% |  |
|  | PBDE-100 in ng/L of plasma | 0% |  |
|  | PBDE-153 in ng/L of plasma | 0% |  |
|  | PBDE-154 in ng/L of plasma | 25% | Imputation to ½ LOD |
|  | PBB-153 in ng/L of plasma | 0% |  |
| PFAS in ng/mL of serum | PBFS in ng/mL of serum | 100% | Elimination |
|  | PFHxS in ng/mL of serum | 0% |  |
|  | PFHpS in ng/mL of serum | 0% |  |
|  | PFOS in ng/mL of serum | 0% |  |
|  | PFDS in ng/mL of serum | 100% | Elimination |
|  | PFOSA in ng/mL of serum | 1% | Imputation to ½ LOD |
|  | N-MeFOSAA in ng/mL of serum | 4% | Imputation to ½ LOD |
|  | N-EtFOSAA in ng/mL of serum | 7% | Imputation to ½ LOD |
|  | PFBA in ng/mL of serum | 100% | Elimination |
|  | PFPA in ng/mL of serum | 100% | Elimination |
|  | PFHxA in ng/mL of serum | 100% | Elimination |
|  | PFHpA in ng/mL of serum | 9% | Imputation to ½ LOD |
|  | PFOA in ng/mL of serum | 0% |  |
|  | PFNA in ng/mL of serum | 0% |  |
|  | PFDA in ng/mL of serum | <1% | Imputation to ½ LOD |
|  | PFUnA in ng/mL of serum | 1% | Imputation to ½ LOD |
|  | PFDoA in ng/mL of serum | 100% | Elimination |

# Supplementary table 2: Loading factors for each substances on each of the 4 components obtained by principal component analysis

| Log-transformed substances | Component 1 | Component 2 | Component 3 | Component 4 |
| --- | --- | --- | --- | --- |
| PBDE-28 | **0.74** | 0.07 | -0.07 | -0.01 |
| PBDE-47 | **0.96** | 0.04 | -0.02 | 0.04 |
| PBDE-99 | **0.91** | 0.03 | 0.03 | -0.06 |
| PBDE-100 | **0.94** | 0.10 | -0.02 | 0.15 |
| PBDE-153 | **0.57** | -0.14 | 0.14 | **0.44** |
| PBDE-154 | **0.80** | 0.06 | 0.06 | 0.09 |
| PBB-153 | 0.09 | -0.08 | 0.09 | **0.75** |
| PFHxS | 0.00 | **0.79** | 0.00 | -0.11 |
| PFHpS | 0.00 | **0.85** | 0.18 | 0.00 |
| PFOS | 0.01 | **0.82** | 0.24 | 0.25 |
| PFOSA | -0.08 | 0.25 | **0.74** | 0.11 |
| N-MeFOSAA | 0.00 | -0.02 | **0.64** | 0.03 |
| N-EtFOSAA | 0.01 | 0.17 | **0.73** | -0.01 |
| PFHPA | 0.18 | 0.37 | 0.40 | 0.01 |
| PFOA | 0.07 | **0.74** | 0.31 | 0.02 |
| PFNA | 0.10 | **0.83** | 0.00 | 0.28 |
| PFDA | 0.07 | **0.58** | -0.04 | **0.66** |
| PFUnA | 0.05 | **0.44** | 0.03 | **0.69** |

*In* ***bold:*** *value >0.40*

# Supplementary table 3: Associations between adherence to PCA components and all breast cancer risk. Components are used in continuous and in quintiles in conditionnal logistic regression models. Odds Ratio (OR) and 95% Confidence Intervals (CI) are presented.

|  | **All breast cancer** | | | |
| --- | --- | --- | --- | --- |
|  | Number (%) - Control | Number (%) - Cases | OR [95% CI] | p-value |
|  | N=194 | N=194 |  |  |
|  |  |  |  |  |
| Component 1, continuous | 194 (100.00) | 194 (100.00) | 0.99 [0.78; 1.25] | 0.9190 |
| Component 1, quintiles |  |  |  | 0.5825 |
| Quintile 1 | 45 (23.20) | 32 (16.49) | Reference |  |
| Quintile 2 | 33 (17.01) | 45 (23.20) | 1.90 [0.83; 4.34] |  |
| Quintile 3 | 40 (20.62) | 38 (19.59) | 1.41 [0.65; 3.08] |  |
| Quintile 4 | 40 (20.62) | 38 (19.59) | 1.26 [0.56; 2.83] |  |
| Quintile 5 | 36 (18.56) | 41 (21.13) | 1.66 [0.75; 3.66] |  |
|  |  |  |  |  |
| Component 2, continuous | 194 (100.00) | 194 (100.00) | 0.93 [0.74; 1.18] | 0.5605 |
| Component 2, quintiles |  |  |  | 0.8668 |
| Quintile 1 | 38 (19.59) | 39 (20.10) | Reference |  |
| Quintile 2 | 43 (22.16) | 35 (18.04) | 0.73 [0.33; 1.59] |  |
| Quintile 3 | 35 (18.04) | 43 (22.16) | 0.83 [0.40; 1.74] |  |
| Quintile 4 | 37 (19.07) | 41 (21.13) | 0.88 [0.40; 1.94] |  |
| Quintile 5 | 41 (21.13) | 36 (18.56) | 0.66 [0.28; 1.53] |  |
|  |  |  |  |  |
| Component 3, continuous | 194 (100.00) | 194 (100.00) | 1.14 [0.90; 1.43] | 0.2788 |
| Component 3, quintiles |  |  |  | 0.0700 |
| Quintile 1 | 45 (23.20) | 32 (16.49) | Reference |  |
| Quintile 2 | 39 (20.10) | 39 (20.10) | 1.04 [0.49; 2.21] |  |
| Quintile 3 | 37 (19.07) | 41 (21.13) | 1.80 [0.81; 3.98] |  |
| Quintile 4 | 29 (14.95) | 49 (25.26) | **2.59 [1.18; 5.72]** |  |
| Quintile 5 | 44 (22.68) | 33 (17.01) | 1.04 [0.47; 2.29] |  |
|  |  |  |  |  |
| Component 4, continuous | 194 (100.00) | 194 (100.00) | 0.95 [0.74; 1.20] | 0.6486 |
| Component 4, quintiles |  |  |  | 0.1379 |
| Quintile 1 | 39 (20.10) | 38 (19.59) | Reference |  |
| Quintile 2 | 39 (20.10) | 39 (20.10) | 1.18 [0.56; 2.47] |  |
| Quintile 3 | 30 (15.46) | 48 (24.74) | 2.21 [0.95; 5.12] |  |
| Quintile 4 | 43 (22.16) | 35 (18.04) | 0.96 [0.43; 2.13] |  |
| Quintile 5 | 43 (22.16) | 34 (17.53) | 0.75 [0.34; 1.66] |  |

All models are adjusted on total plasma lipid content (ng/L, continuous), smoking status (never vs. ever), physical activity measured in metabolic equivalent tasks (MET)-hour/week (continuous), education (≤ 12 years, 12 to 14 years, > 14 years), personal history of benign breast disease (no vs. yes), and family history of breast cancer (none, in first-degree relatives, in extended relatives), parity and age at first fullterm pregnancy (FFTP) (no children, 1 or 2 children and < 30 years old at FFTP, ≥3 children and < 30 years old at FFTP, ≥ 30 years old at FFTP), total breastfeeding duration (never, ≤6 or > 6 months), age at menarche years, continuous), current use of menopausal hormone therapy (yes, no), use of oral contraceptives (ever vs. never), menopausal status and age at menopause (menopause before age 51, menopause at age 51 or later).

# Supplementary figure 1: Correlation matrix between log-transformed exposure to substances in the study population. Pearson’s rank correlation coefficients are presented.

**
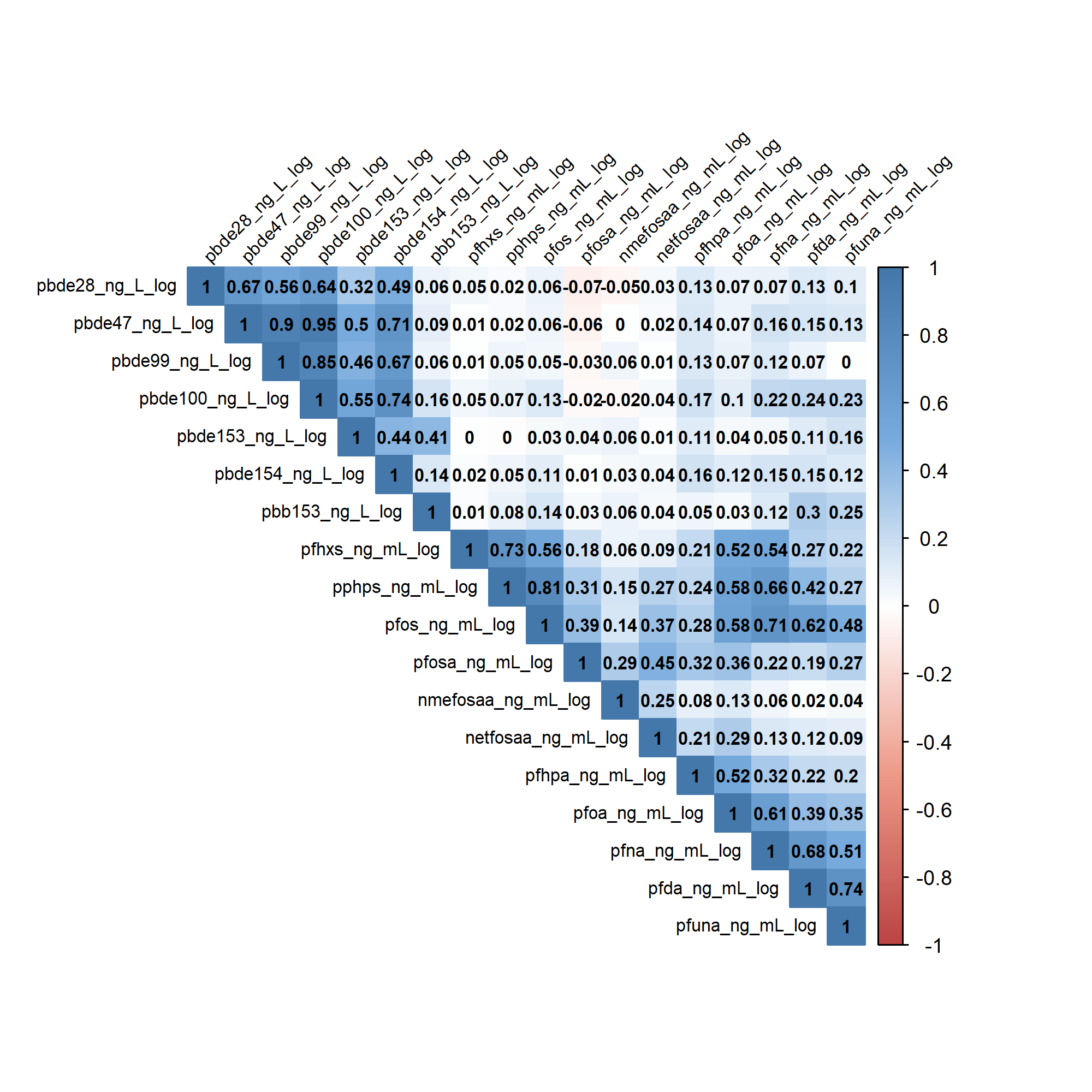
**

# Supplementary figure 2-A: Conditional posterior inclusion probabilities of substances within group for all breast cancer risk.


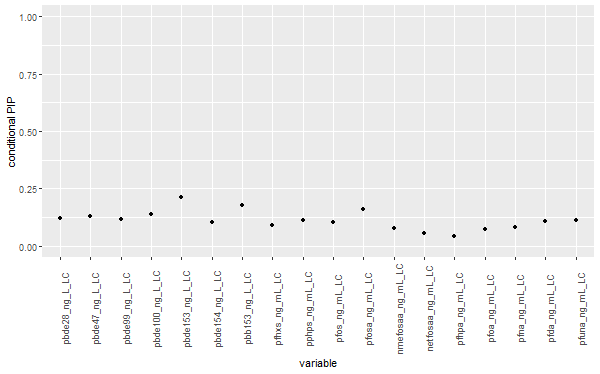


# Supplementary figure 2-B: Conditional posterior inclusion probabilities of substances within group for ER+ breast cancer risk.


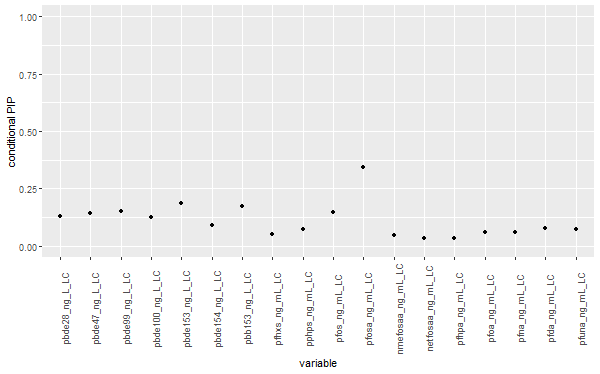


# Supplementary figure 2-C: Conditional posterior inclusion probabilities of substances within group for ER- breast cancer risk.


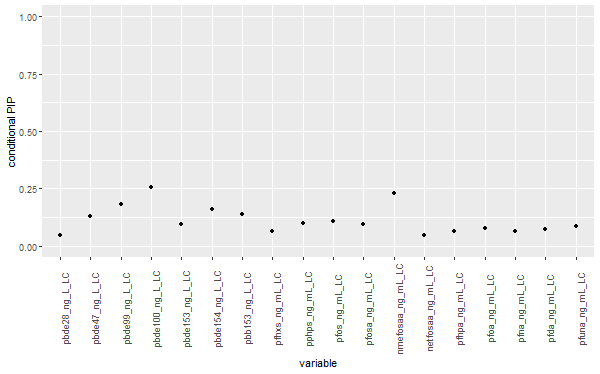


# Supplementary figure 3: Exposure-response functions between exposure to Substance 1 and probit of probability of having a breast cancer while Substance 2 is fixed at defined percentiles (20th, 50th, and 80th), all other substances being fixed at their median value.


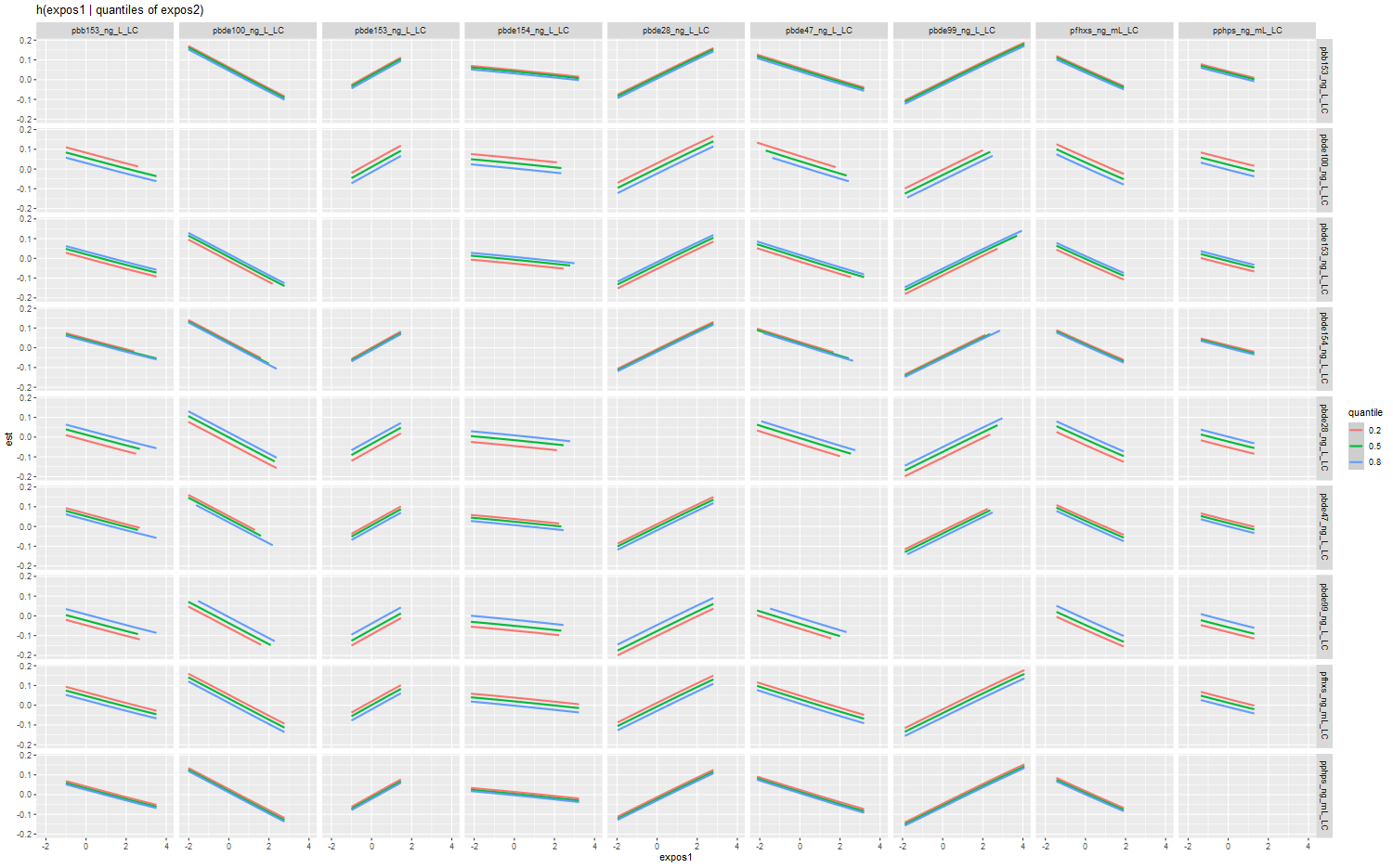


**Supplementary figure 3 (continued)**
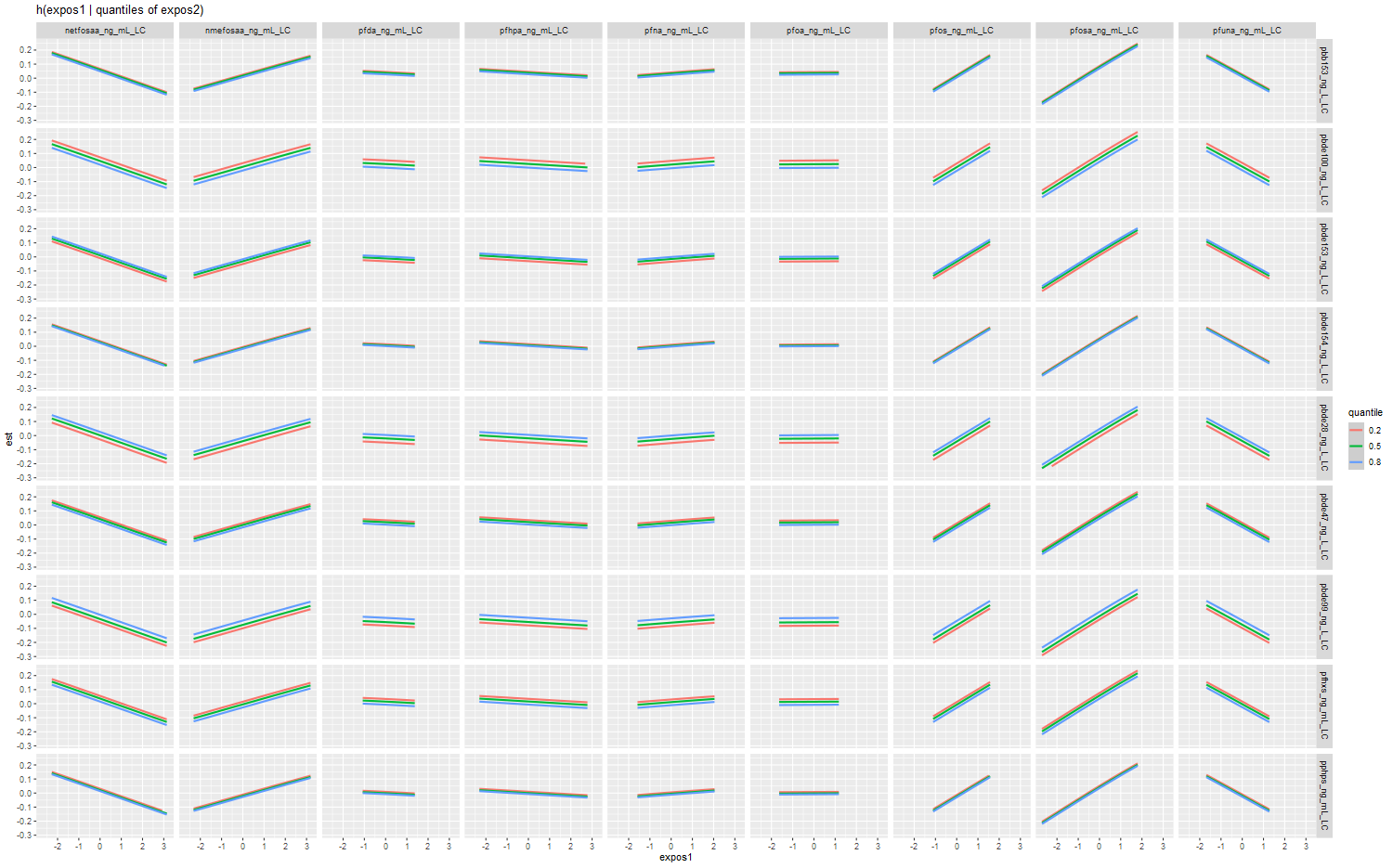


**Supplementary figure 3 (continued)**
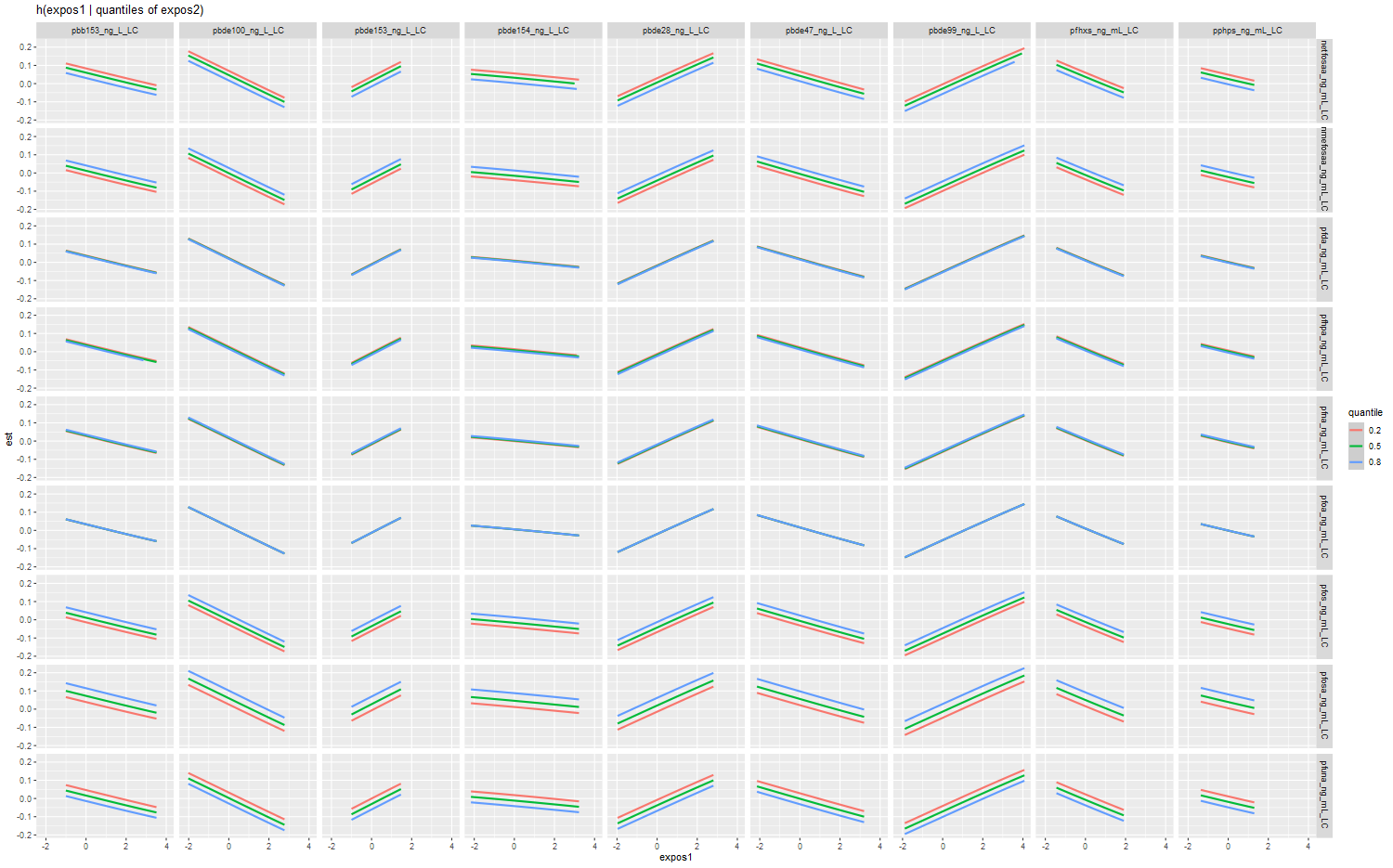


**Supplementary figure 3 (continued)**
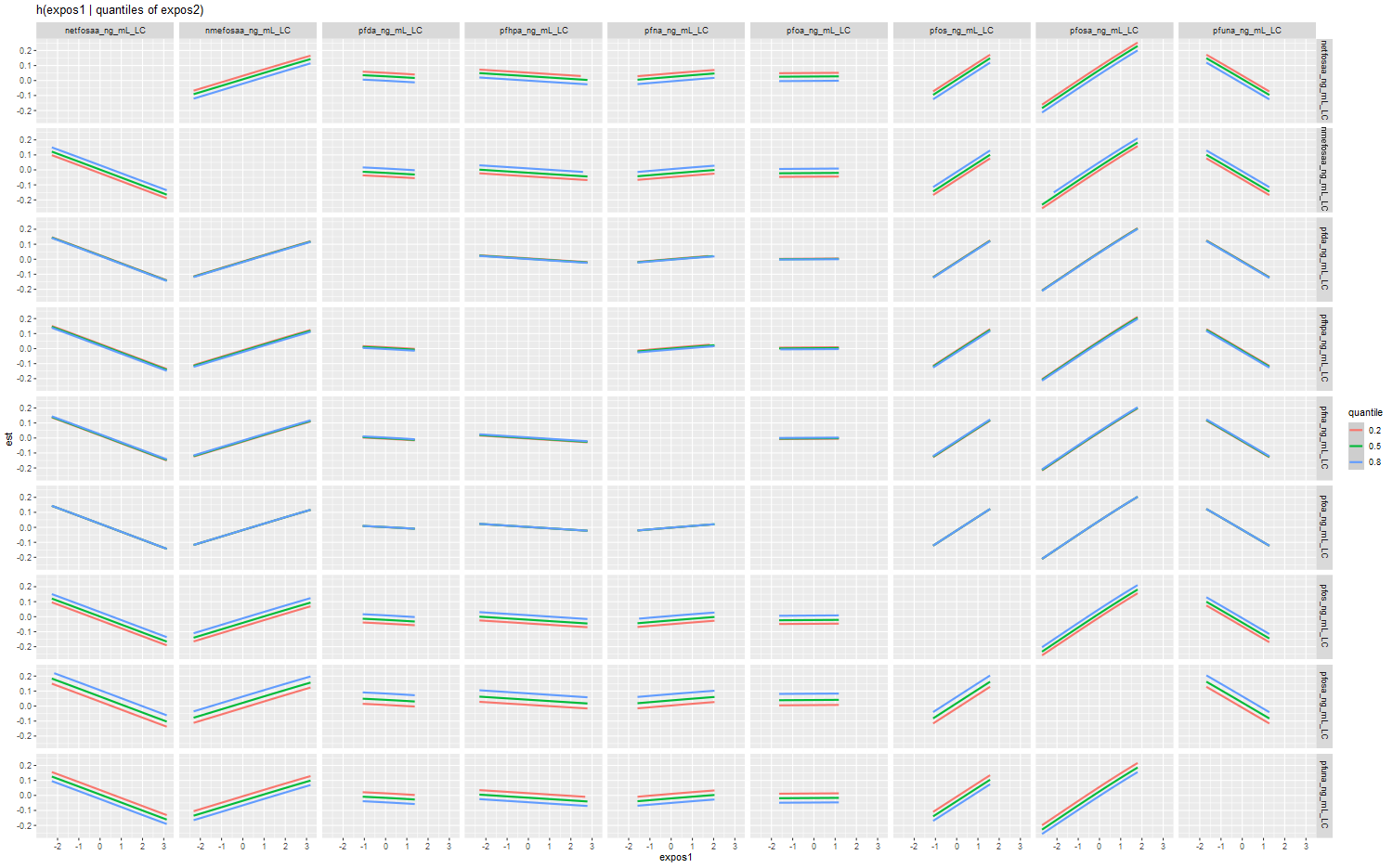


# Supplementary figure 4: Cumulative effect of PFAS, PBB and PBDE for all breast cancer risk.


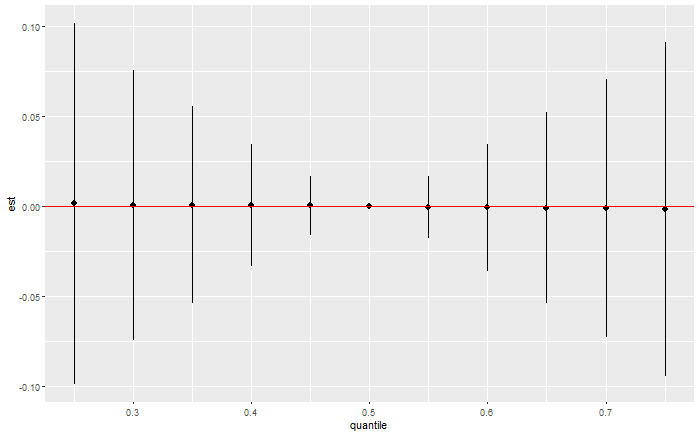

Supplement: Supplementary file 1 — Additional file 1: Table S1. Percentage of values below the Limit Of Detection (LOD) and associated decision for each substance measured in the present study. Table S2. Loading factors for each substances on each of the 4 components obtained by principal component analysis. Table S3. Associations between adherence to PCA components and all breast cancer risk. Components are used in continuous and in quintiles in conditionnal logistic regression models. Odds Ratio (OR) and 95% Confidence Intervals (CI) are presented. Figure S1. Correlation matrix between log-transformed exposure to substances in the study population. Pearson’s rank correlation coefficients are presented. Figure S2-A. Conditional posterior inclusion probabilities of substances within group for all breast cancer risk. Figure S2-B. Conditional posterior inclusion probabilities of substances within group for ER+ breast cancer risk. Figure S2-C. Conditional posterior inclusion probabilities of substances within group for ER- breast cancer risk. Figure S3. Exposure-response functions between exposure to Substance 1 and probit of probability of having a breast cancer while Substance 2 is fixed at defined percentiles (20th, 50th, and 80th), all other substances being fixed at their median value. Figure S4. Cumulative effect of PFAS, PBB and PBDE for all breast cancer risk. [file 12940_2022_840_MOESM1_ESM.docx]
